# Factors and challenges influencing impression management practices among Arab researchers in mass communication on Academic Social Networks

**DOI:** 10.3389/frma.2025.1553049

**Published:** 2025-08-01

**Authors:** Dina Elkhattat, Alyaa Anter

**Affiliations:** ^1^College of Mass Communication, Ajman University, Ajman, United Arab Emirates; ^2^College of Mass Communication, Ain Shams University, Cairo, Egypt

**Keywords:** ASN, SNS, impression management (IM), mass communication, academia

## Abstract

The study aims to explore the motivations and challenges affecting the utilization of Academic Social Networks among Arab mass communication researchers. Identifying the impression management strategies and practices among them and highlighting the obstacles these researchers face. An online survey was applied, collecting a convenience sample consisting of 100 respondents from six Arab countries. Countries include Egypt, Saudi Arabia, the United Arab Emirates, Iraq, Palestine, and Morocco. The study Findings showed no statistically significant differences in the time spent on ASNs based on gender. However, the analysis indicated differences in service usage between males and females. The findings also revealed significant variations in impression management practices based on gender, which highlight concerns regarding gender equality among Arab researchers. The study's findings identified several obstacles that prevent Arab researchers in Mass Communication from fully utilizing academic social networks (ASNs). The respondents cited plagiarism as the most significant concern, with 39%. Findings highlight the need for further studies to explore these barriers. The study also recommends conducting more research studies to compare the influence of disciplines on ASN practices, investigate how the area of specialization impacts research practices by comparing theoretical and applied specializations, and examine how specialization affects the use of ASNs, allowing for a comparison of differences among researchers in the sciences, arts, and humanities.

## Introduction

Social Networking Sites (SNSs) have become a common topic across diverse academic disciplines, including sociology, anthropology, geography, social psychology, organizational studies, and computer science, in recent years (Barnes, 1954). Musial and Kazienko (2013) defined social networks as digital representations of registered users linked by relationships derived from their activities, shared communications, or direct connections within an Internet-based system. The increasing use of information technology and Internet applications in daily life has led to the development of a wide range of social networks. A social network consists of a set of nodes and the links that represent the relationships between these nodes (Rohani and Hock, 2009). In the late 1990s, SNSs and user-friendly publishing tools such as blogs gained popularity, enabling users to publish content and engage in conversations. Technology, especially social media, open-source software, and affordable smartphones, has seen rapid advancements. Moreover, there has been significant growth in broadband network services and multimedia technologies, such as YouTube and podcasts. New tools that bridge the physical and virtual worlds, like augmented reality services and the Internet of Things, have also emerged (Siemens and Conole, 2011). In recent decades, communication and information technology have advanced significantly, leading to widespread use of the Internet and web services (Web 1.0 and Web 2.0) across various fields among institutions and individuals. SNSs have gained popularity among internet users worldwide.

Numerous studies investigated self-impression management to explore how academics and researchers present themselves on SNSs. Veletsianos (2012) examined the practices of researchers on SNSs, focusing on their motivations and concerns regarding these platforms. The study involved 1,372 participants and analyzed 100 tweets from researchers on Twitter. The results indicated that researchers utilized SNS for a variety of reasons, including sharing information, resources, and videos related to their research, seeking assistance from others, offering suggestions to colleagues, communicating lecture details to students, and engaging in social interactions. Moreover, social networks provide a platform for researchers to express their digital identities, manage impressions, exercise self-control, and receive feedback. Madhusudhan (2013) also conducted the use of SNSs by researchers at Delhi University and found that while the majority used these networks mainly for browsing, a smaller group engaged with them for scientific research purposes, focusing on the presentation and promotion of their work. Facebook and ResearchGate^1^ are the most popular platforms for academic purposes among many researchers, primarily because they facilitate communication with peers. However, there are concerns regarding privacy issues and the potential for cyberbullying on these networks. A study by Ovadia (2014) also highlighted the significance of using professional SNSs, such as Twitter and LinkedIn, to manage researchers' online presence and promote their research and professional image. Olufunke et al. (2015) conducted a study on the use of SNSs for academic purposes among 138 academics at Covenant University in Nigeria. The results indicated that 58% of the participants used Social Networking Sites for research purposes, while 31.3% engaged in discussions and collaborated with peers globally. A significant majority of academics, 91.3%, reported that they enjoy using modern technology for their research.

With the advancement of technology, SNS have evolved and diversified into general, professional, and specialized networks. Academic Social Networks (ASNs) began to emerge in the late 1990s, with platforms like ResearchGate, Academia.edu,^2^ and Google Scholar.^3^ These networks quickly became valuable tools for global academic communication and interaction among researchers and academics. They offer a wide range of beneficial services and features that help users showcase their work, manage their professional presence, and engage in research activities. ASNs target the support of researchers' needs. The first type of ASN aims to create profiles and exchange contacts, such as Academia.edu and ResearchGate. The second type focuses on publishing and sharing academic content with other social networking features, such as Mendeley.^4^ This categorization reflects a similar structure seen in general social media (Jordan, 2019). Recently, academic institutions in the Arab region have been using ASNs to enhance their research efforts, improve their reputation, and maintain a high academic ranking among their peers. As a result, academics and researchers are leveraging these networks to manage their professional image, establish connections, and collaborate with peers worldwide. To explore this topic further, the study aims to examine the utilization of ASNs among Arab Mass Communication researchers to identify the level of awareness, motivations, and strategies for managing self-impressions to reflect a positive academic and professional identity and reputation while addressing the obstacles and challenges these researchers face which hinder the effective use of these networks in the Arab world. Ultimately, the goal is to provide recommendations for improving the utilization of ASNs and their services among Arab researchers.

## Literature review

### The importance of ASNs among researchers

During the past decade, several ASNs have gained popularity among researchers, such as ResearchGate, Academia.edu, and Google Scholar. These platforms enable researchers to connect with peers worldwide, share their articles and research, and engage in professional discussions. They have streamlined the exchange of scientific knowledge and expertise by allowing for the immediate publication of research ideas and projects. Additionally, ASNs offer a cost-effective way for researchers to establish their online presence and identity (Dutta, 2010). Moreover, ASNs play a vital role in the information and education industry, facilitating communication, interaction, and collaboration among academic and research communities. Researchers use these platforms to share information, engage with peers, and seek expertise in specific fields, all of which contribute to their professional development (Mohammad et al., 2018).

Numerous studies investigated the importance of ASNs among academics and researchers. Jeng et al. (2015) conducted a case study on the Mendeley website involving 146 academics to examine their use of ASN services. The findings revealed that while academics were more engaged and interactive in academic activities, they were less involved in social activities. Furthermore, users who participated in more groups on the site were more motivated to enhance their professional image and were more likely to share research articles with others. Elsayed (2016) investigated Arab researchers' attitudes and perceptions toward the use of ASNs. The study reveals that the majority of respondents use these networks to share publications, and most researchers subscribed to more than one ASN, but ResearchGate was the most frequently used one. Sheikh (2016) conducted a study on the awareness of ASNs among 516 faculty members at the COMSATS Institute of Information Technology (CIIT) in Pakistan. The findings revealed a high level of awareness among the respondents. Most participants were members of multiple networks, and a significant number had been using these platforms for over 3 years, with LinkedIn being the most frequently used network. The academics expressed a positive attitude toward ASNs, highlighting their benefits in facilitating interactions and discussions with peers and experts in their fields, assisting with research, and promoting research visibility. A study by Duffy and Pooley (2017) explored how individuals use ASNs and compared these behaviors to work patterns in other industries. The findings revealed that media professionals and those in creative fields focus on projecting a unique personal image on these platforms. On the contrary, academics often feel pressured to engage in self-promotion. The study found that the academic environment resembles other social networks in its use of self-promotion, user-created content, and unpaid work based on participation. The utilization of analytics and statistics contributes to a culture of continuous self-monitoring among academics, who are encouraged to track their progress. University policies that measure impact levels reinforced this tendency to overcome potential challenges in academic life.

Al-Daihani et al. (2018) studied the use of SNSs and ASNs among social science academics at Kuwait University, surveying 46 faculty members. The results revealed an increasing use of Facebook and Twitter, followed by Instagram and YouTube. The usage of ASNs ranged from medium to low, with ResearchGate being the most popular platform, followed by Academia.edu. The primary reason for using these networks was to stay connected with the academic community, followed by informal communication with colleagues. Factors that hindered the utilization of these networks included a lack of encouragement from the university and time constraints. Yan et al. (2020) conducted a study on the usage of ResearchGate among researchers from 61 U.S. universities, categorizing them into six groups based on their profile information. Findings showed that scientists in various fields used and interacted with the platform in different ways. For instance, researchers in the social sciences prioritized maintaining a good reputation, while those in the arts and humanities showed lower engagement on the network. Additionally, researchers effectively used ResearchGate to communicate, interact with peers, and participate in discussions. The characteristics of user interaction and usage varied based on specialization, with researchers from universities with higher research activity performing better than those from less research-active institutions.

Mason (2020) investigated the role of ASNs in Japan, noting a decrease in the country's international rankings despite improvements to its education system. One contributing factor is the lack of global engagement among researchers. The study examined the usage of Academia.edu and ResearchGate among researchers from eight universities in Japan. The results indicated a decrease in researchers' reliance on their research activities on Academia.edu, while their engagement with ResearchGate was moderate. Many researchers did not utilize the interactive features of ASNs to connect effectively with researchers worldwide. The study recommended providing training on these network features, highlighting their potential as valuable tools for fostering international academic collaboration and communication. Cozma and Dimitrova (2020) examined the motivations behind the use of ResearchGate among academics in mass communication and their level of satisfaction with the platform. Their findings indicated that academics motivated by external factors to conduct research tend to update their profiles more often but receive fewer benefits from the network. Respondents noted that the influence assigned to researchers on the platform primarily stems from a social system rather than serving as an accurate measure of individual research impact. Assistant professors reported feeling greater social pressure to engage in research activities compared to professors, which led to more powerful external incentives than those experienced by honorary professors. Additionally, doctoral students displayed a higher motivation for research than honorary professors, professors, and assistant professors.

Jordan and Weller's (2018) study of the use of ResearchGate among academics. Findings reported excessive emails, unreliable endorsements, privacy concerns, and a heavier workload. These drawbacks outweigh the advantages, such as user-friendliness and its role in improving academic profile visibility. The findings raised concerns about time management as a primary concern related to these networks, rather than privacy issues or the risks of abuse concerning academic identity. Thelwall and Kousha (2015) examine the utilization of ResearchGate among researchers to disseminate their work. Findings showed that these platforms led to potential changes in the dynamics of informal research communication. Results also revealed a lack of usage in countries such as Brazil, India, China, South Korea, and Russia. These countries seem not to benefit from utilizing ResearchGate to maximize the academic impact of their publications. Table 1 highlights the most famous ASNs among academics and researchers.

**Table 1:** ASNs data based on the official website.

**Networks**	**Launch date**	**Institution**	**Source**
Google Scholar	November 2004	Google	scholar.google.com
ResearchGate	May 2008	ResearchGate GmbH	researchgate.net
Academia	September 2008	Academia Inc.	academia.edu
Mendeley	August 2008	Mendeley Ltd. Elsevier	Mendeley.com
Zotero	October 2006	Corporation for Digital Scholarship	Zotero.org
Frontiers	2007	Frontiers Media SA	frontiersin.org ^5^

### The provided services of ASNs

The ASNs offer their subscribers a variety of services, which include communication, publishing, promotion, and interpersonal interaction. Furthermore, several advanced paid features are available for those who need to benefit from them. Table 2 provides a summary of services and capabilities offered by ASNs.

**Table 2:** Provided Services on ASNs.^6^

**Provided Services**
•Ease and speed of creating free profiles/accounts to present the researchers' identity and manage their impressions.
•Importing user contacts to find and add other researchers besides the network recommendation of researchers with the same research interest.
•Easy and free publishing and promoting research publications and activities among millions of researchers globally.
•Control the availability of shared data with others based on the researchers' desire.
•Diversity of publishing forms: text publications, files, images, documents, links, etc.
•Easy communication and engaging in discussions with peers and experts in the specialty.
•Private interaction through direct messages and public interaction through posts.
•Follow the research activity of other researchers globally to enhance the knowledge about the latest trends and new research areas in the specialty.
•Enhance partnerships and collaborative work by announcing new research ideas and research projects.
•Asking research questions and benefiting from the answers of other researchers and experts globally.
•Notifications through emails to reflect the reading, downloading, citation rates, publications, and achievements of other researchers.
•Motivating, praising, celebrating, and rewarding researchers for their achievements via emails to highlight their progress, or sending them medals upon passing any new level or milestone.
•Display statistics to reflect social and research interaction with the researcher and his publications.
•Spreading knowledge about the latest conferences, grants, and new research projects, and the ability to recommend research reading for other followers.
•Employment services that present job vacancies and fellowships available in the specialty.
•Upgrade account services in exchange for additional paid services for researchers.

### Concept of impression management

Impression management is the process by which individuals attempt to control or influence how others perceive them through the management of their behavior, appearance, and communication. Goffman (1959) was one of the first scholars to explore the concept of self-presentation. He explained that individuals strive to present themselves as acceptable to others. Goffman described various strategies of impression management that people use based on their motivations. Individuals often wear metaphorical masks, play specific roles, and present themselves in ways that align with how they view themselves or how they believe others perceive them. The implementation of impression management strategies for social networking sites depends on their nature, size, and diversity, while audience characteristics affect self-presentation.

Leary (1996) clarified that self-presentation is related to impression management. Self-presentation involves controlling how others perceive individuals during interactions. Schlenker (1980) described it as a series of behaviors used by individuals to create, control, affirm, protect, and enhance the self-image perceived by significant others. According to Gilmore and Ferris (1989) and Bolino et al. (2008), impression management refers to conscious and unconscious efforts to shape and influence the perceptions that others have of us during interactions, which involves presenting oneself in a manner that conveys a positive and acceptable impression and image to others. Bozeman and Kacmar (1997) suggest that most human behavior in organizations and social groups is influenced and driven by factors such as impression management and the desire to be perceived by others in a certain way. The “self-regulation model of impression management processes” explains how impression management works by showing the desired social identity that individuals want to achieve. According to the model, the performer (individual or organization) receives feedback from the targeted audience they are trying to impress regarding the image they aim to convey. The performer continuously compares this feedback with the desired goal. If the comparison reveals that the performer has successfully reflected the desired image to the audience, they will continue to use the same strategies. On the contrary, no discrepancy imposes alternative methods.

### Strategies and motives for impression management on SNSs and ASNs

The approach to managing one's image depends on goals and motivations, which are essential factors in determining how individuals and groups present themselves to others to affect the impressions formed of them (Rui and Stefanone, 2013). Technology has facilitated self-presentation for individuals to present themselves through personal websites, which are efficient tools for self-expression. They can also create personal accounts

on Social Networking Sites to manage their identity and make a positive impression on others (Wilson and Proudfoot, 2014).

Accordingly, E-communication has introduced numerous frameworks for interaction between individuals as an alternative to face-to-face interaction. Goffman focused on the depth and richness of daily interaction in his study. These factors may not occur in electronic interaction while self-expression and its tools remain available. Technology has developed many tools for expression, and as the culture of electronic communication has evolved, individuals now have greater ease and facility in communication and self-expression (Tashmin, 2016). Electronic impression management on social media platforms provides individuals with new tools. Photos, stories, posts, and short videos, besides offering new ways for users to measure the success of their impression management strategies through interaction statistics with the content. Results of Papacharissi's (2002) study showed that online self-presentation was affected by the lack of awareness of the social context and potential audience of posts. To gain broad acceptance from users of these networks, online presenters must carefully control and strategically manage their performance. Hence, managing one's impression online is more complex than face-to-face interaction because it allows for greater control over the desired image. Social networks bridge previously separate social groups and prompt users to share messages since individuals actively manage and control the impressions that others form of them. They can use E-communication tools for self-presentation and impression management. Moreover, Zhang's (2024) study revealed that students frequently portray different personas online rather than in real life, influenced by factors such as audience segregation, privacy concerns, and conformity. Most of their posts focus on positive experiences, with common impression management strategies being idealization and mystification. Therefore, the study recommended that SNS developers improve privacy and audience segregation features to better cater to user needs.

Lee et al. (1999) defined self-presentation tactics as behaviors used to manage impressions to achieve short-term personal goals or objectives. The tactics include defensive and assertive strategies. Defensive strategies are applied when something threatens or harms the image. The goal is to restore the image or lessen the negative impact of these consequences. Assertive strategies are proactive behavioral efforts that involve creating and maintaining a specific image. Rui and Stefanone (2013) identified strategies for managing individuals' accounts on social networks when publishing to diverse and heterogeneous audiences, suggesting that individuals should use private communication channels and prevent specific members from viewing content. Moreover, they can categorize their network members into different groups to reduce the problems of conflicting social domains for users.

**The motives for impression management on social networks include the following:**

- **Social motives**: According to Leary and Kowalski (1990), individuals sometimes manage impressions and control their behavior to achieve social goals. Schlenker (1980) also suggests that social motive involves the expected value approach to assess the individuals' image value, which they need to convey to others to receive social rewards, such as acceptance, friendship, support, and assistance.
- **Personal motives**: According to Krämer and Winter (2008), personal motives affect the efforts to maintain self-esteem. Individuals are motivated to enhance their self-esteem by making a positive impression on others. Crabtree and Pillow (2017) identified motives to belong and build social relationships, self-presentation, and the perception of network density, which reflect the awareness of using social networks to achieve impression management. Dominick (1999) conducted the strategies or tactics identified by Jones and Pittman (1982) from offline communication to the online environment. **These five strategies include:**

° **Ingratiation**: individuals use this tactic to appear attractive and likable through praise, flattery, humility, humor, understanding, warmth, agreement, and compassion.
° **Exemplification**: Individuals use this tactic to seem morally superior by showing ideological commitment, fighting for a cause, making sacrifices, demonstrating self-discipline, and volunteering their time to help others. Those who employ this tactic do more than necessary to appear superior or devoted to a noble cause.
° **Self-promotion**: Individuals use this tactic to demonstrate competence, qualifications, and competitiveness. It includes claims related to abilities, achievements, performance, qualifications, and expertise highlights.
° **Intimidation or threat**: Individuals use this tactic to gain power through dominance threats, expressions of anger, resentment, and challenging competitors.
° **Supplication**: Individuals use this tactic as a communication strategy to appear helpless and ask for others' assistance. It includes making requests for help and self-denial, admitting a lack of solutions, and expressing the need for assistance (Jones and Pittman, 1982).

We can conclude that ASNs are similar to SNSs, but they specifically target academics and researchers. ASNs are free platforms that enable users to create profiles, connect with other researchers, and share content related to academic topics. One of the most appealing features of these sites for researchers is the ease of use and publishing services for articles and scientific work. Researchers can easily connect and share with colleagues and scientific communities worldwide (University of Toronto, 2020). In this study, impression management refers to the researchers' intentional use of tools, methods, and strategies for self-presentation purposes on Academic Social Networks to positively influence their image and how others perceive them, ultimately helping them build a positive academic reputation.

### Obstacles and challenges that hinder the utilization of ASNs

Many studies raise challenges and obstacles that may hinder the best utilization of ASNs among users. Köchling (2025) highlighted several challenges related to copyright infringement, as publishers like Elsevier deleted uploaded papers due to violations of copyright agreements, which raises concerns about the stability of these platforms as archives for scholarly content. Jordan and Weller (2018) also highlighted digital illiteracy, privacy and security, and the reliability of online information as significant barriers to researcher participation and interaction with ASNs. Additionally, Moran et al. (2011) noted that many academics are worried about privacy and integrity issues on SNSs. Gu and Widén-Wulff's (2011) findings highlighted that copyright issues become complicated in the online sphere, and it is difficult to evaluate the reliability of information. Findings also demonstrated intellectual property concerns and the potential for plagiarism among most researchers, who fear sharing their research on these networks.

Furthermore, previous studies demonstrated multiple concerns and obstacles that may hinder the best utilization of ASNs among scholars. Some studies focused on the sociological dimension and its relation to the impact of these networks on impressions. Köchling's (2025) results revealed that junior researchers face increasing pressure to be active on these platforms to gain visibility and enhance their reputation. On the contrary, inactive ones may face ignorance by the academic community. Jordan and Weller's (2018) study also reported that using these networks may increase workloads and reduce academic freedom, potentially putting additional pressure on academics. Some other studies raise concerns about the accuracy of measuring the impact on social media platforms.

Thelwall and Kousha's (2015) findings highlighted that social media may exacerbate existing inequalities and biases in academia, with some researchers having more visibility and influence than others. Ali et al. (2017) also mentioned the role of the algorithm and the Academic Social Networks scores, driven by an opaque algorithm based on member activity, publications, and reads. This algorithm may prioritize metric optimization over research quality. The lack of correlation between RG scores and institutional rankings undermines their reliability and usefulness as a sole indicator of research quality. Köchling (2025) also showed concern about relying on opaque algorithms to regulate visibility, collaboration, and evaluation, as it threatens academic equality and transparency. Chaudhuri and Baker (2018) identified challenges in ASN site usage, including low faculty representation, time-consuming profile identification, a mismatch between user numbers and document uploads, and limited engagement with institutional repositories. Finally, Köchling (2025) highlighted the commercialization risk of scholarly communication, as the platforms monetize user data and metadata, raising concerns about their alignment with scientific missions. The boundaries between formal and informal scholarly communication are becoming blurred, transforming traditional practices and increasing the demand for continuous engagement. These challenges underscore the tension between the platforms' stated goals of supporting science and their commercial interests. It is important to investigate the challenges and obstacles that hinder Arab Mass Communication researchers in this study.

## Research methodology

Recently, academic institutions in the Arab region have been using ASNs to enhance their research efforts, improve their reputation, and maintain a high academic ranking among their peers. As a result, academics and researchers are leveraging these networks to manage their professional image, establish connections, and collaborate with peers worldwide. To explore this topic further, a descriptive study was applied using survey methodology to examine the motivations and challenges affecting the use of ASNs among Arab researchers in mass communication. The study also aims to identify the impression management strategies and practices that help reflect a positive academic and professional identity and reputation while addressing the obstacles these researchers face.

### Sampling and data collection

The study population consisted of Arab Mass Communication researchers who had an Academic Social Network account during the implementation period, ensuring they were familiar with self-promotion techniques on this platform. Researchers determined convenience sampling as the most suitable type for the study. This approach represents a non-probability sampling method involving respondents who were easily accessible and relevant to the research topic (Galloway, 2005). Accordingly, an online questionnaire invitation was distributed via hyperlinks on SNSs and ANSs to collect the data. During the implementation, the data collection process faced challenges because researchers sent 200 invitations and only received 100 responses during the implementation period. The response rate was 50%, likely due to the multiple duties and responsibilities these researchers have, such as research, teaching, and thesis supervision. The characteristics of the study sample are demonstrated in Table 3. The sample includes participants from six Arab countries: Egypt, Saudi Arabia, the United Arab Emirates, Iraq, Palestine, and Morocco. Participants' contributions varied across many government, private, and higher institutions.

**Table 3:** Characteristics of the study sample.

**Variables**	**Categories**	**%**
Gender	Female	54
	Male	46
Age	25–34	48
	35–44	36
	45–54	14
	55 and above	2
Specialization	Radio and television	45
	Public relations and advertising	22
	Journalism	20
	New media	12
	General mass communication	1
Degree/Title	Professor	8
	Associate Professor	10
	Assistant Professor	48
	Teaching assistants	14
	Researchers (Master's - PhD)	20
Employer	Governmental	54
	Private Sector	32
	Independent	4

A total of 25 institutions from Egypt participated in the survey, including 12 governmental universities. Ain Shams University, Cairo University, Al-Azhar University, Beni Suef University, Menoufia University, Sohag University, Benha University, Minya University, Mansoura University, Suez University, Zagazig University, and Alexandria University. Additionally, respondents participated from five private universities, including Sinai University, Pharos University, Misr University for Science and Technology, Al-Ahram Canadian University, and 6th of October University. There were also participants from eight higher academic institutions. Alexandria Higher Institute of Media, Al-Jazeera Higher Institute of Media and Communication Sciences, International Academy of Engineering and Media Sciences, International Higher Institute of Media in Shorouk, Higher Institute of Media and Communication Arts, Shorouk Academy, Maritime Academy, and Canadian International Media Institute. Respondents from various Arab countries participated in the study, including Palestine, Palestine Technical College, Al-Aqsa University, Al-Istiqlal University, Gaza University, and the Islamic University of Gaza, Saudi Arabia “Imam Muhammad bin Saud Islamic University and Taibah University” United Arab Emirates “Ajman University and the Emirates College of Technology” Iraq “University of Baghdad” Morocco “King Mohammed VI University” Additionally, researchers from the Arab Open University and the League of Arab States also participated in the study.

The e-questionnaire was divided into two sections to meet the study's objectives. The first section comprised eight questions focused on researchers' habits, practices, motivations, usage, reliance on Academic Networking Services (ANSs), and strategies for impression management. The second section contained six questions designed to gather demographic information, including gender, age group, specialty, academic degree, workplace, and nationality. The data were analyzed using the Statistical Package for the Social Sciences (SPSS).

## Proposed model, research questions, and hypothesis

### Proposed model

Rosenberg (2009) outlines four strategies and dimensions for managing online impressions, including:

- Manipulation: This strategy involves using negative tactics, such as threats and supplication, to influence perceptions.
- Damage Control: This defensive approach involves justifying actions, offering apologies, and denying or justifying negative actions.
- Self-Promotion: This strategy emphasizes showcasing one's abilities and achievements to enhance one's image.
- Role Modeling: This involves engaging in behaviors that are admired by others, utilizing techniques such as ingratiation, flattery, exemplification, and presenting oneself as a moral and dedicated role model.

Uziel (2010) suggested redefining scales that measure impression management as self-regulation scales. This redefinition could help identify individuals who demonstrate high levels of self-regulation, especially in social contexts. In contrast, manipulation indicates a lack of self-regulation, which can negatively impact an individual's self-image and relationships with others. Furthermore, Nadia et al. (2020) found a correlation between deceptive self-presentation and poor mental health, while Mun and Kim (2021) highlighted that deceptive self-presentation is related to increased levels of depression among users.

The researchers employed this theoretical framework to investigate ASNs, which provide various features and services for managing impressions. These include profile pictures, biographies, research posts, publishing and promoting research, and providing research recommendations. Additionally, the statistics on user interactions through these accounts and the content shared present a valuable opportunity to assess the effectiveness of methods used for self-presentation, the formation of academic identity, and impression management among researchers.

### Impression management scale

**Figure 1:**
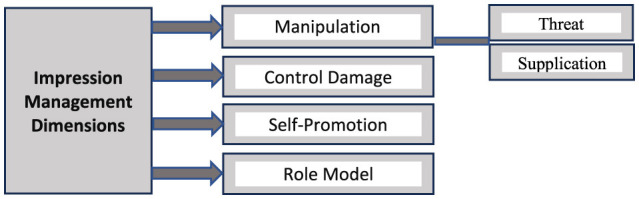
Prepared by researchers based on the Rosenberg (2009) scale.

The study aimed to investigate impression management strategies among Arab researchers in Mass Communication using an Electronic questionnaire incorporating the Modified Self-Presentation Tactics Scale, developed by Lee et al. (1999) and Rosenberg (2009). This scale comprises four dimensions, as illustrated in Figure 1. The researchers adapted and modified the scale to be relevant to electronic self-presentation on ASNs. Notably, they excluded the manipulation strategy, as it reflects negative practices that do not apply to impression management on ASNs used by researchers. This particular strategy involves deceit, which contradicts research values and ethical self-regulation, and individuals seldom admit to using such tactics.

This study employed a scale consisting of 13 statements designed to measure three dimensions of electronic impression management and self-presentation: Self-Promotion, Damage Control, and Role Modeling. Respondents rated these statements using a four-point Likert scale (1–4), which reflects their behaviors and practices related to impression management strategies on ASN accounts. To assess the scale's validity and reliability, the Split-Half method was applied. The results yielded a high-reliability coefficient of 0.93, indicating that the scale is dependable in measuring the intended constructs. More details regarding the scale's validity and reliability can be revealed in Tables 4, 5.

**Table 4:** Validity and reliability of the impression management scale on ASNs.

**Correlation coefficient**	**Odd**	**Even**
Correlation coefficient (Pearson)	1	0.929^**^
(2-tailed)	100	0.000
*N* (sample size)		100
Correlation coefficient (Pearson)	0.929^**^	1
(2-tailed)	0.000	100
*N* (sample size)	100	

^**^The test indicates a high reliability coefficient.

**Table 5:** The scale validity and the correlation of items with the overall score.

**Strategy**	**Statements**	**Item-total correlation**
Self-promotion	1. I am keen to upload and update my data on ASN.	0.775
	2. I care about uploading my picture on ASN.	0.696
	3. I upload my full research publications to ASN.	0.788
	4. I share comprehensive information about my interests and experience.	0.833
	5. I announce new research projects with my colleagues.	0.865
	6. I pay attention to the statistics of my followers on ASN.	0.807
Control damage	7. I apologize for not sharing the research and explaining the reasons.	0.836
	8. If I respond late to inquiries, I explain the reasons to reduce negative impressions.	0.839
	9. I apologize for comments that may hurt another researcher.	0.786
Role model	10. I care about communication with peers globally to exchange experiences.	0.868
	11. I provide some comments and suggestions to other researchers.	0.873
	12. I pay attention to praising researchers to gain their support and assistance.	0.803
	13. I recommend reading others' research.	0.834

### Research questions

Q1- What is the knowledge source of ASN among Arab Mass Communication Researchers?
Q2- What are the Arab Mass Communication researchers' motives for using ASNs?
Q3- What is the usage rate of ASNs among Arab Mass Communication researchers?
Q4- How do Arab Mass Communication researchers benefit from the services of ASNs?
Q5- What strategies are used by Arab researchers to manage their presence and impressions of ASNs?
Q6- What obstacles affect Arab Mass Communication researchers in using ASNs?

### Hypotheses

H1- There are significant differences in the usage rate of ASNs according to gender and age group.
H2- There are significant differences in the reliance on ASNs' services according to gender and age group.
H3- There are significant differences in the Practice of Impression Management Strategies on ASNs according to gender and age group.

## Results and findings

***Q1- What is the knowledge source of ASN among Arab Mass Communication*** ***Researchers?***

Table 6 demonstrates that 97% of survey respondents use ASNs, indicating a growing interest in these platforms within the Arab region. This interest is high due to the networks‘ significance in enhancing academic reputation and rankings. Additionally, ASNs play a vital role in promoting universities, research institutions, and individual researchers. Respondents cited various sources of information about ASNs, with search engines being the primary source at 48%. Furthermore, 17% of respondents noted that their employers influence their awareness of these networks, reflecting institutions' current efforts to encourage members to create ASN accounts as part of their professional responsibilities. The impact of SNSs and friends ranked third, with 13% of respondents acknowledging that their peers have helped raise awareness of ASNs. Friends often promote their research through personal accounts or ASNs, effectively managing their professional image and increasing citations of their work. These findings align with Sheikh's (2016) study, which indicated that most researchers learned about ASNs through online searches.

**Table 6:** Sources of knowledge about ASN among Arab researchers.

**Sources**	**%**
Search engines	48
Employer	17
Friends	13
Social networks	13
Other sources: research footnotes - by chance	6
Don't know ASN well	3
Total	100

### ASNs knowledge resources according to age group

The study examined the relationship between different age groups and the sources of knowledge among Arab researchers regarding ASNs. The findings presented in Tables 7, 8 revealed that researchers under 35 years old obtain information about these networks from a variety of sources. Search engines ranked the highest at 50%, followed by the workplace at 41% and social networks at 38.5%. Search engines were the most significant source of knowledge by 40% for the age group between 35 and 44 years. The workplace followed closely at 41%, while social networks and friends each contributed 38.5%. Researchers aged 45–55 made up 14% of the total sample; for this group, the primary source of knowledge about these networks was also search engines, with social networks and friends each representing 23%. Senior researchers aged 55 and above comprised 2% of the sample. The principal sources of knowledge for the two respondents were workplace interactions and colleagues. That emphasizes the importance of workplace interactions, which play a crucial role in encouraging senior researchers to use these networks.

**Table 7:** ASNs knowledge resources by age group.

**Age group**	**Search engines**	**SNSs**	**Employer**	**Friends**	**Accidentally**	**Occasionally**	**Total**
	**%**	**%**	**%**	**%**	**%**	**%**	**%**
−25	50	38.5	41.2	30.8	100	66.7	48
−35	39.6	38.5	41.2	38.5	0	0	36
−45	10.4	23.1	11.8	23.1	0	33.3	14
+55	0	0	5.9	7.7	0	0	2

**Table 8:** ASNs knowledge resources by academic ranks.

**Academic ranks**	**Search engines**	**SNSs**	**Employer**	**Friends**	**Accidentally**	**occasionally**	**Total**
	**%**	**%**	**%**	**%**	**%**	**%**	**%**
Teaching Assistant	35.4	30.8	23.5	30.8	50	66.6	34
Assistant Professor	45.8	38.5	64.7	53.8	50	0	48
Associate Professor	8.3	23.1	5.9	7.7	0	33.3	10.0
Professor	10.4	7.7	5.9	7.7	0	0	8.0

### ASNs knowledge resources according to the academic ranks

Additionally, the workplace played a significant role in encouraging researchers from various academic ranks to create accounts on ASN, promoting their research, enhancing their university's ranking, and improving their institution's reputation among competitors.

***Q2- What are the Arab Mass Communication researchers' motives for using*** ***ASNs?***

Table 9 shows the reasons and the motives behind the use of ASNs. Being updated with the latest trends in the field was the first motive, cited by 66%. Followed by the desire to communicate with peers in their specialization, cited by 57%. Additionally, the study found that employers have a significant influence on researchers' participation in ASNs, ranking third in motives at 45%. This trend could be beneficial for both government and private organizations, as it plays a vital role in ranking and promoting universities. Moreover, it promotes the advancement of research conducted by university members, enhancing the institution's reputation. Respondents express their interest in the influence of ASNs on their professional reputation and increasing visibility. The motivation to utilize impression management on ASNs ranked fourth at 26%, demonstrating a strong interest in developing a professional image. Moreover, there was a notable interest in the services offered by these networks. The desire to obtain jobs and research grants ranked fifth at 25%, followed by the pursuit of research partnerships at 15%. The interest in general knowledge and technology services is at 3%. Although these later percentages are lower compared to the top motivations, they indicate that a segment of researchers is benefiting from the advanced services provided by these networks. These users are not solely browsing or publishing; instead, they are actively engaging in research communication and forming partnerships with peers worldwide, which are essential features of these networks.

***Q3- What is the usage rate of ASNs among Arab Mass Communication*** ***researchers?***

**Table 9:** Respondents' motives for subscription on ASNs.

**Motives**	**%**
Follow up with the latest trends in the specialty	66
Connect with other researchers in the specialization	57
Employer requirements to create accounts on ASNs	45
Self-promotion and building a positive mental image	26
Acknowledge the job vacancies and research grants	25
Building connections with new research partnerships	15
Other: Keeping up to date with the latest technology, following researchers, and references	3

### Utilization rate of ASNs among Arab academics and researchers

The study aimed to measure the utilization of ASNs among Arab researchers and their habits on these networks by monitoring three key factors: the percentage rate of use, the number of years they have been using them, and the average time spent on these networks. The findings revealed that a significant number of respondents use ASNs either regularly or occasionally. Among the networks surveyed, Google Scholar ranked first, with 90% of respondents indicating its use; ResearchGate followed in second place at 71%, and Academia ranked third at 70%. This outcome contrasts with the findings reported by Mohammad et al. (2018), which showed that ResearchGate had the highest usage rate compared to other networks. Additionally, Table 10 indicates that several ASNs are not well-known among respondents. The study found that 64% of respondents were unfamiliar with the Frontiers website, 62% were unaware of Zotero,^7^ and 53% were not familiar with Mendeley. This lack of recognition may stem from insufficient promotion and the limited services offered by these ASNs compared to the top three networks.

**Table 10:** Preferences of ASNs among respondents from Arab academics and researchers.

**Networks**	**Always**	**Sometimes**	**Total users**	**Know but not a user**	**Don't know**	**Non-users**
	**%**	**%**	**%**	**%**	**%**	**%**
Google Scholar	56	34	90	7	3	10
ResearchGate	34	37	71	12	17	29
Academia	27	43	70	15	15	30
Mendeley	4	9	13	34	53	87
Zotero	2	6	8	30	62	92
Frontiers	2	7	9	27	64	91

**Figure 2:**
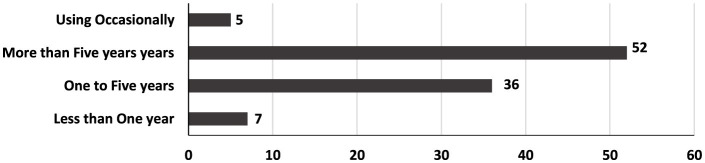
Utilization of ASNs among Arab academics and researchers in mass communication.

According to Figure 2, more than half of the study participants have been using ASNs for over 5 years, while 36% had used them for a period of 3–5 years. This trend reflects a growing awareness among Arab academics and researchers of the importance of ASNs in shaping a positive professional image and promoting their published research. Additionally, many academic institutions encourage their members to create profiles on these platforms to enhance their university's reputation and improve its visibility and ranking. These findings are consistent with previous studies that have highlighted the recent increase in the use of these networks.

**Figure 3:**
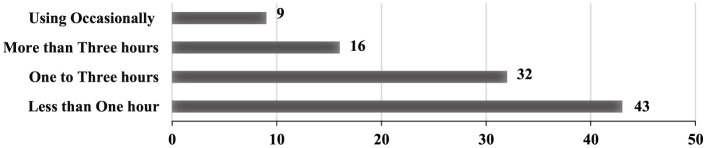
Average number of times spent on ASNs.

Despite a high percentage of respondents using ASNs, Figure 3 demonstrates that their usage time is relatively short. Specifically, 43% of respondents reported using these networks for less than an hour per day, 32% used them for 1–3 h daily, and only 16% engaged with these networks for more than 3 h per day. This low usage rate may be due to researchers focusing primarily on their work. Many tend to use ASNs briefly to publish their research or stay updated on their interests. Consequently, the findings suggest that Arab academics and researchers are not fully leveraging these networks for scientific research. There is a significant need to raise awareness and encourage them to utilize these platforms more effectively.

***H1- There are significant differences in the usage rate of ASNs according to*** ***gender and age group:***

#### Significance of differences in the usage rate of ASNs by gender

An analysis of the time spent on ASNs by gender revealed no statistically significant differences. As shown in Table 11, both males and females spend a similar amount of time on these networks. This similarity may stem from the shared demands of research work and the increasing expectation from employers for academics and researchers to maintain ASN accounts. This trend has led to a more equal usage of these networks between genders.

**Table 11:** Significance of differences in the usage rate of ASNs by gender.

**Variable**	**Female (*****N*** = **54)**		**Male (*****N*** = **46)**		***T-*** **value**	**Significance**
	**Average**	**Standard deviation**	**Average**	**Standard deviation**		
Number of hours used on ASNs	1.48	0.86	1.63	0.88	0.853	**X**

#### Significance of differences in the usage rate of ASNs by age group

The study aimed to compare researchers based on age groups by dividing them into two groups. The first one is for participants below 35 years, and the second is for those 35 years and above. According to the data presented in Table 12, there were no statistically significant differences between the two age groups in the time spent on ASNs. The findings indicated that the amount of time spent on these networks did not vary significantly by age, and both younger and older researchers found them to be beneficial and user-friendly.

**Table 12:** Significance of differences in the usage rate of ASNs by age group.

**Variable**	**Less than 35 years**		**More than 35 years**		***T-*** **value**	**Significance**
	***N*** = **48**		***N*** = **52**			
	**Average**	**Standard deviation**	**Average**	**Standard deviation**		
Number of hours used on ASNs	1.56	0.897	1.54	0.851	0.138	X

Regarding **Hypothesis 1**, the analysis supports the null hypothesis, indicating no significant differences in the time spent on ASNs between different age groups or genders.

***Q4- How do Arab Mass Communication researchers benefit from the services*** ***of ASNs?***

The data in Table 13 indicate a high usage rate of ASN services among Arab researchers. Respondents highlighted the significance of these networks for staying updated on the latest research in their fields (96%), accessing full references (93%), sharing research ideas and projects (92%), and promoting their work to enhance citation rates (77%). These findings align with Veletsianos' (2012) study, which underscores the role of ASNs in helping researchers establish their digital identity and manage their online presence. However, findings showed lower usage of interactive services among Arab researchers for communication, collaboration with peers, consultations, discussions, and partnerships. Additionally, many Arab researchers do not best utilize the services to upgrade their accounts.

**Table 13:** Utilization rate of ASN services among Arab academics and researchers.

**Statements**	**High**	**Medium**	**Low**	**Users**	**Using occasionally**	**Average of usage**	**Standard deviation σ**
	**%**	**%**	**%**	**%**	**%**		
View the latest research in the specialty	62	30	4	96	4	32	27.54
Selecting and announcing new research ideas and projects	38	42	12	92	8	30.67	17.47
Facilitating communication, collaboration, and research in companies	24	27	25	76	24	25.33	1.414
Promoting published research and increasing citations	32	23	22	77	23	25.67	4.690
Learning researchers' opinions about research work	19	32	21	72	28	24	6.05
Benefiting from scientific consultations in the specialty	25	26	22	73	27	24.33	2.160
Benefiting from references and published research	62	27	4	93	7	31	26.69
Searching for job opportunities and research grants	15	25	24	64	36	21.33	8.602
Putting research questions and discussions, and collecting feedback	17	28	21	66	34	22	7.527
Learning about research performance indicators reports	18	27	20	65	35	21.67	7.702
Upgrading membership for additional paid services	10	16	28	54	46	18	15.87

### Utilization rate of ASN services among Arab academics and researchers by year

The data presented in Table 14 indicates that over half of the total respondents have used ASNs for more than 5 years. Additionally, 36% of researchers reported using these networks for a duration ranging from 1 to 5 years, while only 7% have been users for less than a year. The results demonstrate similar usage patterns among these three groups. The most frequently used services included reviewing the latest trends in their field, obtaining references, generating new ideas, and promoting innovative projects. This outcome reflects the importance of these networks in providing valuable services to researchers, which led to continued usage of ASNs. On the contrary, interest in interactive services has decreased compared to research services. Fewer users are looking to upgrade their accounts for additional features. Users who have been with the service for more than 5 years are the most likely to utilize these advanced services. This trend highlights and clarifies the relationship between the length of time a user has been active and their willingness to adopt advanced features that enhance their research activities and solidify their identity with these accounts.

**Table 14:** Utilization rate of ASN services among Arab academics and researchers by years.

**ASNs services**	**Usage rate by years**					
	**Less than a year**	**1–5 Years**	**More than 5 Years**	**Using occasionally**	**Non-users'**	**Total**
	**%**	**%**	**%**	**%**	**%**	**%**
View the latest research in the specialty	100	100	98.1	100	0	96
Announce new research ideas and projects.	100	94.4	96.2	50	0	92
Communication, collaboration, and research partnerships	85.7	75	78.8	100	0	76
Promote published research and increase citations	85.7	80.6	76.9	100	0	77
Collect feedback from other researchers about research projects.	71.4	75	75	50	0	72
Benefit from scientific consultations in the specialty	85.7	72.2	75	100	0	73
Benefit from published references and research	85.7	94.4	81.1	100	0	93
Search for job opportunities and research grants	57.1	63.9	69.2	50	0	64
Ask and collect research questions and discussions.	71.4	58.3	75	50	0	66
Learn about research performance indicators reports	71.4	63.9	69.2	50	0	65
Upgrade membership for additional paid services	57.1	47.2	61.5	50	0	54

***H2- There are significant differences in the reliance on ASNs' services*** ***according to gender and age group**.*

#### Significance of differences in the degree of reliance on ASNs according to gender

An analysis of ASN service usage by gender revealed statistically significant differences at the 0.01 level. In Table 15 females showed an average reliance percentage of 15%, while males had an average of 21%. This discrepancy may occur because women often juggle multiple family responsibilities alongside their work and research commitments, leading to lower usage rates compared to men, which raises concerns about gender equality among Arab researchers, particularly considering various cultural and social factors, as highlighted in some studies.

**Table 15:** Significance of differences in the degree of reliance on ASNs according to gender.

**Variable**	**Female (*****N*** = **54)**		**Male (*****N*** = **46)**		***T-*** **value**	**Significance**
	**Average**	**Standard deviation**	**Average**	**Standard deviation**		
Degree of reliance on ASNs' services	15.06	7.88	20.91	8.12	3.653	0.01

Jonsson's (2024) study highlights how cultural and social contexts influence women's perceptions of entrepreneurship. The findings showed that the power individuals possess significantly influences these perceptions, especially in individualistic cultures where people depend on their judgments. Moreover, the findings suggest that women's participation in entrepreneurship tends to increase in liberal environments that foster high gender equality and emphasize individualistic values. Bührer et al. (2020) proposed an assessment framework to examine the impact of flagship programs in Germany on publications, excellence rates, and their contributions to achieving gender equality. The findings indicate that these programs increased the representation of women in research organizations, improved the quantity and quality of publications, and enhanced their citation rates.

#### Significance of differences in the degree of reliance on ASNs according to the age group variable

The data presented in Table 16 revealed no statistically significant differences in reliance on ASN services between age groups. The researchers from both age groups are using ANS' services similarly, as they engage in the same tasks and activities in their research work.

**Table 16:** Significance of differences in the degree of reliance on ASNs according to age group.

**Variable**	**Less than 35 years**		**More than 35 years**		***T*** **-value**	**Significance**
	***N*** = **48**		***N*** = **52**			
	**Average**	**Standard deviation**	**Average**	**Standard deviation**		
Degree of reliance on ASNs' services	16.40	08.042	19.00	8.749	1.546	X

Regarding **Hypothesis 2**, the analysis revealed statistically significant differences at the 0.01 level in the reliance on ASNs based on gender. While supporting the null hypothesis, indicating no significant differences in the degree of dependency on ASNs based on age groups.

***Q5- What strategies are used by Arab researchers to manage their presence*** ***and impressions of ASNs?***

Table 17 indicates a significant decrease in the use of impression management strategies by Arab researchers through ASNs. The respondents showed interest in several self-promotion strategies, role models, and damage control techniques. The most commonly used self-promotion strategy was the keen interest in uploading and updating account information, with an average usage rate of 28%. Followed by a 24% rate for uploading personal photos to profiles and a 23.6% interest in sharing research and teaching interests and experiences. These results suggest that institutions are encouraging researchers to create and maintain their accounts to reap mutual benefits.

**Table 17:** Strategies for Arab academics and researchers to manage ASN accounts.

**Strategy**	**Statements**	**Usage rate**			**Average usage**	**Using occasionally**	**Standard deviation σ**
		**High**	**Medium**	**Low**			
		**%**	**%**	**%**	**%**	**%**	
Self-promotion	I am keen to upload and update my data on ASN.	21	41	22	28	16	10.9848
	I care about uploading my picture on ASN.	13	28	31	24	28	8.12404
	I upload my full research publications to ASN.	14	25	28	22.33	33	8.04156
	I share comprehensive information about my interests and experience.	20	26	25	23.67	29	3.74166
	I announce new research projects with colleagues.	9	18	29	18.67	44	15.0776
	I pay attention to followers' statistics on ASNs.	17	24	26	22.33	33	6.58281
Damage control	I apologize for not sharing the research and explaining the reasons.	17	29	16	20.67	38	10.4881
	If I respond late to inquiries, I explain the reasons to reduce negative impressions.	18	24	20	20.67	38	9.0185
	I apologize for comments that may hurt another researcher.	21	22	13	18.67	44	13.2916
Role model	I care about communication with peers globally to exchange experiences.	22	25	22	23	31	4.24264
	I provide some comments and suggestions to other researchers.	23	22	21	22	34	6.0553
	I pay attention to praising researchers to gain their support and assistance.	11	24	27	20.67	38	11.1056
	I recommend reading others' research.	14	29	26	23	31	7.61577

Researchers were interested in practicing role model strategies, particularly in research communication and collaboration. They reported engaging with peers at a rate of 23% and providing comments and writing suggestions for others at a rate of 22%. Interest in complimenting and praising other researchers to gain their support was notably lower, with an average of only 20.6%. These findings reflect a desire among researchers and academics to communicate and collaborate effectively with their peers and to assist the students they supervise. In contrast, the average percentage of researchers employing damage control strategies decreased compared to the two previous ones. Additionally, the results indicated that Arab researchers in Mass Communication are hesitant to announce their new research projects or publish papers on ASNs due to fears of plagiarism. As a result, their publishing activity has been low despite its importance for self-promotion and the ranking of their institutions.

The findings align with the study of Duffy and Pooley (2017), which showed that ASNs are similar to SNSs in terms of self-promotion goals. They also highlighted that academics feel pressured to engage in self-promotion practices. Similarly, Huang (2014) discovered that self-presentation on SNSs corresponds with traditional self-presentation skills used in face-to-face interactions. Generally, individuals who present themselves online have control over the information they share and can use various strategies to craft their image.

***H3: Significance of differences in the Practice of Impression Management*** ***Strategies on ASNs according to gender and age variables:***

#### Significance of differences in the practice of impression management strategies on ASNs according to gender

An analysis of impression management practices among ASN users reveals significant differences based on gender, with results showing statistical significance at the 0.01 level. As illustrated in Table 18, the average percentage for females is 12%, while for males, it is 21%. This disparity may be attributed to the multiple responsibilities that women often balance at home, at work, and within the realm of scientific research, which could lead to a reduced tendency to employ these strategies on such networks. This result is consistent with Peng's (2020) study, which indicated that academic women have lower research productivity compared to their male counterparts, and there are significant gender differences in domestic publications among them. Various factors, including workplace conditions and family responsibilities, influenced the research productivity of academic women. To address these challenges, they employed strategies such as seeking support from colleagues and joining online communities.

**Table 18:** Significance of differences in the practice of impression management according to gender.

**Variable**	**Female (*****N*** = **54)**		**Male (*****N*** = **46)**		***T*** **-value**	**Significance**
	**Average**	**Standard deviation**	**Average**	**Standard deviation**		
Impression management strategies	12.37	11.04	21.13	10.50	4.043	0.01

#### Significance of differences in the practice of impression management strategies on ASNs according to the age group

The analysis of the age group variable's significance in impression management practices within ASNs revealed no statistically significant differences between the two age groups, as indicated in Table 19. This finding suggests that researchers in both age groups have similar practices, as they share the same research responsibilities and objectives.

**Table 19:** Significance of differences in the practice of impression management according to the age group.

**Variable**	**Less than 35 years**		**More than 35 years**		***T*** **-value**	**Significance**
	***N*** = **48**		***N*** = **52**			
	**Average**	**Standard deviation**	**Average**	**Standard deviation**		
Impression management strategies	15.44	11.566	17.29	11.683	0.795	X

Concerning **Hypothesis 3**, the analysis revealed statistically significant differences at the 0.01 level in the Practice of Impression Management on ASNs between different genders. While supporting the null hypothesis, indicating no significant differences in the Practice of Impression Management on ASNs among various age groups.

***Q6- What obstacles affect Arab Mass Communication academics and*** ***researchers in using ASNs?***

As shown in Table 20, the results indicated that 67% of respondents from Arab Mass Communication academics and researchers encounter obstacles and challenges when using ASNs. The primary challenge identified is the fear of scientific plagiarism, which affects 39% of respondents. The second major obstacle is a preference for face-to-face collaboration, reported by 24% of those surveyed. These findings align with the study conducted by Jordan and Weller (2018), which highlighted digital illiteracy, privacy and security concerns, and the reliability of online information as significant barriers to researcher participation and interaction with ASNs. Additionally, Moran et al. (2011) noted that many academics are worried about privacy and integrity issues on SNSs.

**Table 20:** Obstacles Affecting the Use of ASNs.

**Obstacles**	**%**
Fear of scientific plagiarism	39
Preference for face-to-face collaboration	24
Lack of usefulness	18
Scientific journals copyright	16
Lack of time and Laziness to manage the accounts	4
Total	100

## Discussion

The study collected data from 100 Arab Mass Communication researchers using a convenience sample. Respondents belong to both governmental and private sectors across six Arab countries, including Egypt, Saudi Arabia, the UAE, Iraq, Palestine, and Morocco. The data collection utilized an E-questionnaire via both SNSs and ASNs. Data analysis revealed that 97% of respondents used ASNs, and approximately half of them had been using these networks for at least 5 years. Search engines were the primary source of knowledge about ASNs, accounting for 48%, followed by employers, which represented 17%. The motivations for using ASNs also differ. The most common motive is to stay updated on recent research trends, cited by 66% of respondents, followed by the desire to connect with peers (57%) and employer requirements (45%). Google Scholar emerged as the most popular platform, used by 90% of respondents, followed by ResearchGate and Academia.edu. These findings highlight the differences in users' preferences based on origin, time of services, and motivations, as noted in previous studies, such as Mohammad et al. (2018), which found that ResearchGate was the most utilized network. Al-Daihani et al. (2018) showed the increasing use of ResearchGate as the most used platform, followed by Academia.edu. The motives behind this usage were staying in touch with the academic community, followed by informal academic communication with peers. Sheikh (2016) demonstrated that LinkedIn ranked first with the highest usage rate based on its benefits in interaction, discussions, and promoting research.

The study underscores the perceived value of ASNs and the essential services they provide, particularly in offering access to the latest specialized research. However, it also noted a low response rate of Arab researchers to activities aimed at managing their professional impressions. Researchers expressed their interest in employing role model strategies, especially in research communication and collaboration, to engage with their peers. Cozma and Dimitrova (2020) also investigated the motivations behind the use of ASNs among mass communication academics and their satisfaction. Results revealed that academics with external motivations to conduct research update their accounts more frequently. Assistant professors also felt higher social pressure to pursue their research than professors. Yan et al. (2020) also conducted a study among researchers in the U.S. Results revealed that researchers from social science prioritized maintaining a good reputation, while users in the arts and humanities showed low engagement on the network. Duffy and Pooley (2017) examined the behavior of ASNs, and results showed that media professionals and people in creative fields focus on creating a unique image of themselves on these platforms, while academics feel pressure to engage in self-promotion. Jeng et al. (2015) monitored the use of ASN services. Results showed that academics were more engaged and interactive in academic affairs and less involved in social activities. Users who joined more groups were more motivated to focus on their professional appearance and more likely to share research articles with others.

The study Findings also showed no statistically significant differences in the time spent on ASNs based on gender (H1). However, the analysis indicated differences in service usage between males and females (H2). Maybe because women often balance multiple family responsibilities alongside their work and research duties, which could result in lower usage rates. After examining the differences in impression management practices based on gender, the results revealed significant variations, likely due to similar underlying reasons. The findings highlight concerns regarding gender equality among Arab researchers, particularly in light of various cultural and social factors identified in earlier research. Battaglia et al. (2020) also focused on gender-based discrimination. The study revealed women's low representation in senior leadership positions and scholarly productivity despite research output being crucial for both academic and leadership roles. The study indicated a significant gender-based disparity in leadership positions and academic ranks.

The study's findings identified several obstacles and challenges that prevent Arab researchers in Mass Communication from fully utilizing ASNs. The respondents cited plagiarism as the most significant concern, with 39%. Additionally, 24% expressed a preference for face-to-face collaboration, while only 4% indicated that a lack of time for managing their accounts on these networks was an issue. Consequently, Arab researchers in Mass Communication are hesitant to announce new research projects or publish papers on ASNs due to these concerns. Therefore, their publishing activity remains limited, even though such activities are crucial for self-promotion and the ranking of their institutions. This result is consistent with many previous studies, such as Köchling (2025), Jordan and Weller (2018), Moran et al. (2011), and Gu and Widén-Wulff (2011), which raise concerns regarding intellectual property, integrity, privacy, security, copywriting violations, potential plagiarism, stability of scholarly content as archives, and the reliability of online information.

These results oppose other previous studies that identified different concerns. For example, Köchling (2025) and Jordan and Weller (2018) found that social factors play a significant role in the utilization of ASNs, as they may limit individual freedom while increasing the pressure and workload needed to improve visibility and reputation. Other studies, such as Thelwall and Kousha (2015), Ali et al. (2017), Köchling (2025), and Chaudhuri and Baker (2018), raise concerns about equality and transparency in research quality assessment, the accuracy of measuring the research impact and the biases related to visibility and opaque algorithms scores in prioritize metric optimization. Finally, Köchling (2025) highlighted the commercialization risk of scholarly communication, which raises concerns about their alignment with scientific missions. The boundaries between formal and informal scholarly communication are becoming blurred, transforming traditional practices and increasing the demand for continuous engagement. These challenges underscore the tension between the platforms' stated goals of supporting science and their commercial interests.

## Conclusion and recommendations

In recent decades, communication and information technology have advanced significantly, leading both individuals and institutions to utilize the Internet and various web services (1.0 and 2.0) across different fields. As a result, the types of SNSs have diversified, and ASNs have gained popularity among users due to the growing focus on the reputation of academic institutions and their relationship with university rankings at both local and international levels. Consequently, more Arab academics and researchers recently created ASN accounts because they were encouraged by their employers to utilize these platforms consistently, which heightened the importance of this study to examine their awareness of using ASNs, determine how they manage their impressions on these networks, identifying the motivations and strategies they employ in managing their online presence and self-impression on these platforms, and list barriers and challenges that prevent Arab Mass Communication researchers from effectively utilizing these networks.

The results showed no statistically significant differences in the time spent on ASNs based on gender (H1). However, the analysis indicated differences in service usage between males and females (H2). Maybe because women often balance multiple family responsibilities alongside their work and research duties, which could result in lower usage rates. After examining the differences in impression management practices based on gender, the results revealed significant variations, likely due to similar underlying reasons. However, the analysis indicated no statistically significant differences in the degree of reliance on ASN services and impression management practices across different age groups. This result highlighted that Arab researchers in Mass Communication from different ages utilize ASNs in the same way. This finding refers to the similarity in research responsibilities and objectives (H3).

The study highlights significant concerns regarding gender equality among Arab researchers, particularly in light of various cultural and social factors identified in earlier research. This finding highlights the need for further studies to explore these barriers. Additionally, it addresses the issue of fear of plagiarism, which may deter Arab researchers from publishing on these networks. Moreover, the study suggests improving and enhancing communication and research practices on ASNs among Arab researchers and academics of Mass Communication. Academic organizations should play a vital role in encouraging them to create and manage their accounts on ASN effectively and organize training workshops to explain its features, services, and importance, as well as help them to benefit from the advanced features, services, and inform them about strategies for managing impressions on these networks and heighten their importance for them and the institution and active researchers on these networks can also train their peers. Academic organizations should also provide incentives, such as recognizing and honoring the most active accounts on these networks or establishing a specialized department to monitor their accounts and provide support if needed. Organizations should raise awareness among researchers about the importance of copyrights in protecting their research contributions. Both journals and organizations need to examine submitted research publications for plagiarism to safeguard the rights of researchers against violations. Furthermore, the study suggests that future research should analyze the accounts of ASN's Arab researchers and monitor their practices. The study also recommends conducting more research studies to compare the influence of disciplines on ASN practices, investigate how the area of specialization impacts research practices by comparing theoretical and applied specializations, and examine how specialization affects the use of ASNs, allowing for a comparison of differences among researchers in the sciences, arts, and humanities.

Furthermore, researchers should be encouraged to utilize services on ASNs to develop research partnerships and promote cross-cultural communication with researchers worldwide. This approach can significantly improve research quality. Employers also should play a crucial role by encouraging and supporting collaborative projects that involve multinational teams, which can enrich research endeavors. They can further promote collaboration by providing funding for research projects and incentivizing researchers to engage through these networks.

## Data Availability

The raw data supporting the conclusions of this article will be made available by the authors, without undue reservation.

## Footnotes

^1^ResearchGate (2022). Available online at: https://www.researchgate.net/.

^2^Academia (2022). Available online at: http://academia.edu/.

^3^Google Scholar (2022). Available online at: https://scholar.google.com/.

^4^Mendeley (2022). Available online at: https://www.mendeley.com/.

^5^Frontiersin (2022). Available online at: https://www.frontiersin.org/about/mission.

^6^Prepared by the researchers based on monitoring the provided services on ASNs.

^7^Zotero Website. Available online at: https://www.zotero.org/support/credits_and_acknowledgments.
